# Australian School Student Perceptions of Effective Anti-tobacco Health Warnings

**DOI:** 10.3389/fpubh.2018.00297

**Published:** 2018-10-17

**Authors:** Aaron Drovandi, Peta-Ann Teague, Beverley Glass, Bunmi Malau-Aduli

**Affiliations:** Discipline of Pharmacy, College of Medicine and Dentistry, James Cook University, Townsville, QLD, Australia

**Keywords:** smoking reduction, public health, health behavior, health promotion, adolescent health

## Abstract

**Background:** Recent research posits that anti-tobacco health warnings on cigarette packaging may gradually lose their effectiveness in dissuading adolescents from tobacco products several years after implementation. Health warnings on individual cigarette sticks represent a novel warning medium, and may further educate adolescents on the dangers associated with smoking, and reduce tobacco experimentation amongst this vulnerable population.

**Methods:** In an online survey of school students in Queensland, Australia, participants were requested to rate (on five-point Likert scales) and comment on the perceived effectiveness of current cigarette packaging warnings, and 12 text warnings on cigarette sticks, in preventing non-smokers from smoking, and encouraging current smokers to quit. The warnings were divided into four themes to establish the most effective types of anti-tobacco messages: mortality statistics, health condition consequences, social and financial consequences, and supportive messages. These themes were based on current anti-tobacco interventions within Australia, and the rising cost of tobacco products, and designed to align with the Health Belief Model.

**Results:** Participants (*N* = 150; Age = 15–18) from five schools completed the survey, and generally viewed current packaging warnings as gross and disgusting, and rating them as somewhat effective in preventing non-smokers from smoking. Current warnings were however considered less effective in prompting current smokers to quit with participants describing them as being un-relatable to teenagers, and smokers as having become desensitized to the warnings used. One theme of cigarette-stick warning (mortality statistics) was rated as significantly more effective (*p* < 0.001) than current cigarette packaging, with an odds ratio (OR) of 2.77 (95% confidence interval [CI]: 1.67–4.62). Overall, warnings were considered to be 4.71 times (95%CI: 2.72–6.43, *p* < 0.001) more effective on non-smokers than on smokers. Over three-quarters of participants supported using health warnings on individual cigarette sticks.

**Conclusions:** Current cigarette packaging warnings have retained some effectiveness in dissuading adolescents from smoking, though novel and thought-provoking text-only warnings on cigarette sticks may serve as an additional intervention in reducing tobacco use. Further research requires identification of the most effective warnings, and the perceptions of a more diverse participant base.

## Introduction

Experimenting with tobacco products during adolescence increases the likelihood of developing long-term nicotine addiction, with the majority of active adult smokers having started using tobacco before the age of 20 years (1). Tobacco experimentation at this age often occurs as a result of cigarette sharing in social settings (2), which can lead to a quick loss of autonomy, and addiction occurring more rapidly, and with lower levels of consumption compared to adults (3, 4). This is theorized to occur as a result of an increased disruptive effect of nicotine on brain function within the maturing adolescent brain (5). Given the global mortality rate of an estimated 7 million deaths per year attributable to tobacco use, preventing smoking uptake during this vulnerable period is imperative in improving the health of future generations (6).

Adolescent experimentation with tobacco products is influenced by their limited experience and understanding of the nature of addiction, and their beliefs in being personally able to avoid or control addictive behaviors at will (7, 8). This is in spite of their awareness of the general addictive potential of nicotine, and smoking as being a leading cause of death (9). Their misconceptions on the consequences of tobacco may be in part due to a lack of exposure to informative cigarette packaging health warnings, which are being adopted by over 100 countries as part of the World Health Organization's Framework Convention on Tobacco Control (10). The practice of cigarette sharing amongst adolescents results in a reduction in exposure to tobacco packaging interventions, inhibiting the viewing frequency and effectiveness of these interventions (11–14). Whilst initially effective, recent research has also identified that packaging warnings may lose their effectiveness and impact on health-related decisions and behaviors through repeated exposures amongst both adolescents and adults (15–18).

Factors influencing these key health-related decisions and behaviors are described in the Health Belief Model (19), and includes multiple individual-specific elements. In relation to tobacco use, this includes a person's perceived susceptibility and severity of potential smoking-related consequences, the benefits and barriers to smoking and to quitting, their self-efficacy in doing so, and the cues which prompt smoking, or facilitate quitting. These elements are influenced by knowledge of the positive and negative consequences of each of these decisions. A novel anti-tobacco public health intervention being investigated is the use of health warnings and messages on individual cigarette sticks (20–24). There have only been a handful of studies investigating the potential effectiveness of a small number of cigarette stick warnings (20–24), including smoking kills, minutes of life lost, and the names of carcinogenic cigarette constituents. They found that these warnings reduced cigarette attractiveness, cigarette uptake, and increased quit intentions, with a recent systematic review stating this as an understudied area with further exploratory research needed (25).

It is expected that this form of intervention would both compensate for the lack of warning exposure from cigarette sharing, and supplement current anti-tobacco interventions such as cigarette packaging warnings and mass media campaigns, thus enhancing reader knowledge and improve on the health-related decisions and behaviors of both adults and adolescents. These warnings may increase the perceived threat of cigarette use and their susceptibility in suffering a resulting medical illness, and increase their self-efficacy in avoiding these threats. Similar to the effects of cigarette packaging, this may lead to reductions in tobacco experimentation for non-smokers (particularly adolescents), serve as a barrier to relapse for ex-smokers, and a facilitator of quit attempts for current smokers (26, 27).

This study aims to first investigate adolescents' perceptions toward current cigarette packaging warnings, and their effectiveness in dissuading adolescents from using tobacco products. We also aimed to investigate the potential effectiveness of cigarette stick warnings in educating adolescents on the dangers associated with tobacco use, by gauging their perceptions of how an expanded set of these messages might prevent non-smokers (especially adolescents like themselves) from smoking, and prompt current smokers to quit. Finally, we aimed to identify adolescent support for or against the inclusion of health warnings on individual cigarette sticks.

## Methods

### Study design

This study utilized an online survey of mixed-methods (concurrent triangulation; which allows the use of quantitative and qualitative methods of data collection together to cross-validate findings and overcome weaknesses present in individual methods) design, distributed to private schools in Queensland in November 2017, who approved the research and forwarded the survey link to parents of eligible students. Students in Grades 10, 11, and 12 (aged 15–18 years old) were eligible for participation, with parents (due to ethical requirements) being responsible for discussing participation with the students, and allowing access to the link if they approved participation. Parents were also responsible for emailing the principal investigator if they wanted their child to go into the draw to win one of the $10 Woolworths e-gift vouchers available as an incentive for participation (Woolworths is an Australian retail chain).

### Procedure and data items collected

**Figure 1:**
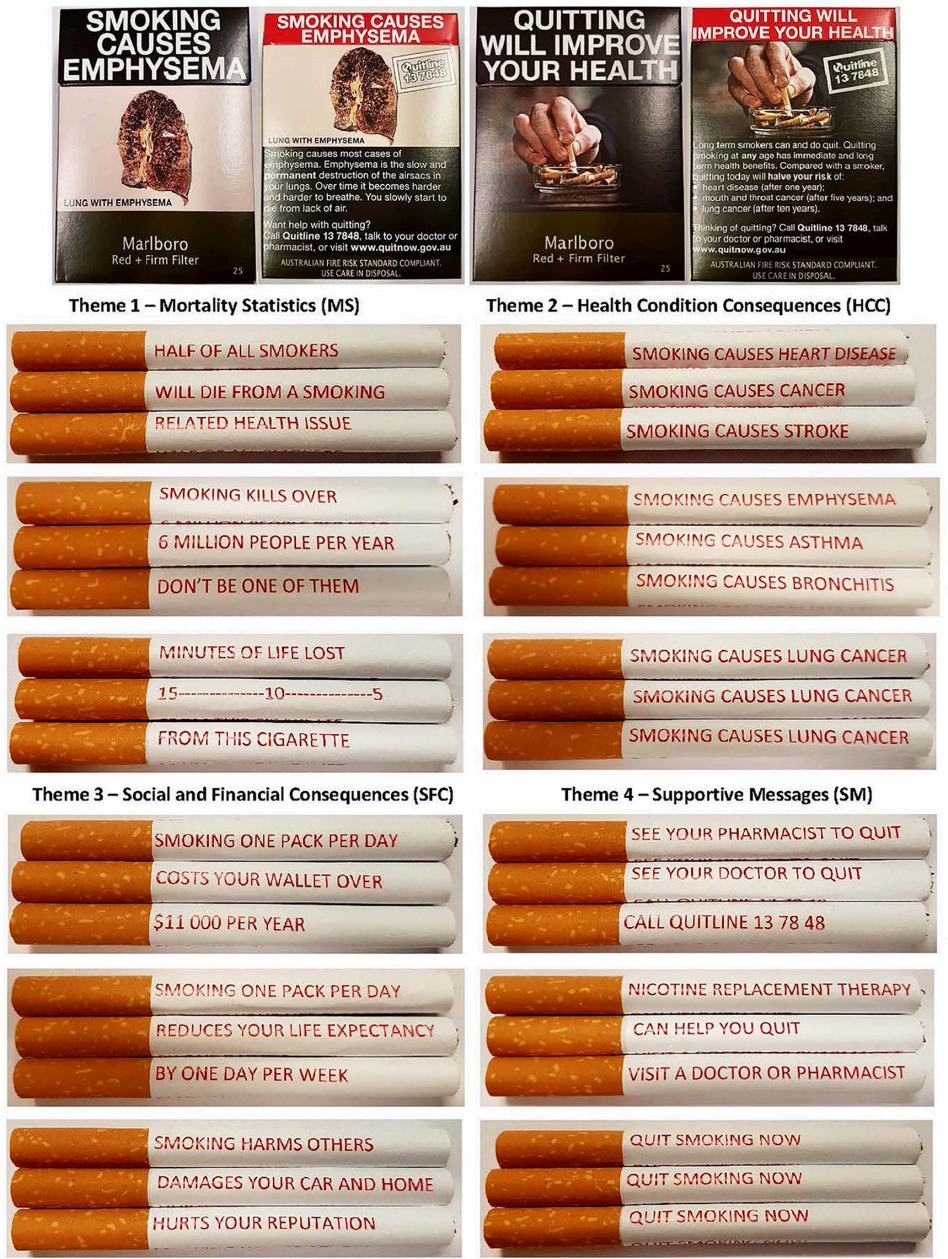
The front and back of two cigarette packaging in circulation in Australia, and the 12 cigarette warnings divided in to the four themes. Each cigarette includes three lines of text and is rotated to read the entire message.

Initial demographic information obtained from participants included: age, gender, grade at school, school attended, and ethnic background. Pre-intervention questions were then presented, with participants first rating on a five-point Likert scale (from not at all harmful to very harmful) their perceptions of how harmful smoking is to a person's health. This was followed by pictures of two of the fourteen current cigarette packaging warnings in circulation in Australia (see Figure 1); one displaying a lung with emphysema, and one encouraging smokers to quit. Eleven of these current packaging warnings in Australia (including the lung with emphysema) describe a negative health aspect of smoking, two describe the effects of smoking on others, and one encourages current smokers to quit. The packaging warnings chosen were representative of the themes of warnings in rotation in Australia at the time of the study. Participants rated on a five-point Likert scale (from not at all effective to very effective) their opinions of the effectiveness of the cigarette packaging warnings in preventing non-smokers from smoking, and prompting current smokers to quit. Each question had optional open-text boxes participants could use to include details relating to their chosen response on the Likert scale. Participants were then given the option to discuss their perceived strengths or shortcomings of current health messages and warnings. They were also given the option to detail any anti-tobacco messages or warnings, either on cigarette packaging, or elsewhere that they considered to be effective or memorable as anti-tobacco interventions.

Photos of 12 cigarette sticks with messages printed in red down their shafts were then displayed. Each cigarette had three lines of text, which can be read as the cigarette is rotated, depicting a full message or warning relating to tobacco use. The cigarettes were grouped into four themes, which were displayed on a single page in a standardized order (see Figure 1): mortality statistics (MS; theme one), health condition consequences (HCC; theme two), social and financial consequences (SFC; theme three), and supportive messages (SM; theme four) to quit smoking. The warnings within themes two and four were chosen to align with current packaging warnings, theme one warnings were an extension of previous research into cigarette-stick warnings and current media campaigns, and theme three as a continuation of the current Australian tobacco climate, with increased stigma toward smokers, and soaring tobacco prices through heavy taxation (28). For each theme, participants rated on a five-point Likert scale how effective (from not at all effective to very effective) they thought each message theme would be in discouraging non-smokers from smoking, and on a second five-point Likert scale on effectiveness in encouraging current smokers to quit. Each cigarette per theme was labeled A, B, and C to allow participants to include comments on individual warnings in optional open-text boxes. Lastly, participants rated on a five-point Likert scale their opinion (from strongly disagree to strongly agree') on the inclusion of health warnings on individual cigarettes.

### Analysis

We first ran a descriptive analysis to determine the characteristics of the study population. Non-parametric tests (Kruskal-Wallis and Mann-Whitney U) were used (SPSS v24; IBM Corp, Armonk, NY, USA) to investigate the relationships between the demographic variables in relation to participant perceptions of the anti-tobacco health warnings, with *p*-value limits of 0.05. Friedman Test was used to measure change in participants' perceptions across the five categories (current warnings and the four interventional themes). *Post-hoc* tests and Bonferroni adjustments were used to determine statistically significant differences between the categories. A random intercepts mixed-effects proportional odds logistic regression was performed using R (v33.2.4; R Core Team, Vienna, Austria) ordinal statistical package (with respondent ID as a random effect, and age group, ethnicity, gender, smoking status, and themes as fixed effects), to evaluate between and within-theme effectiveness (in comparison to current packaging warnings) in dissuading non-smokers and smokers from smoking. Responses from open-text comments were analyzed independently by two authors (AD and BMA) using thematic analysis (NVivo v11; QSR International Pty Ltd, Melbourne, Australia) to confirm emerging themes. To establish trustworthiness of the qualitative data, findings were compared and conflicting interpretations were resolved through dialogue. Illustrative quotes are reported verbatim to support the discussion.

## Results

### Demographic profile

From the five participating schools, 150 students completed the survey. Their demographic characteristics and baseline perceptions of the harms of smoking are shown in Table 1. Most participants (88.0%) resided in the South-East corner of Queensland (which accounts for two-thirds of the state's population), with the remainder residing in Central and North Queensland.

**Table 1:** Demographic characteristics and baseline perceptions of survey participants.

	***N***	**%**
**Gender**		
Male	54	36.0
Female	96	64.0
**Age (years)**		
15	20	13.3
16	74	49.3
17	48	32.0
18	8	5.3
**Grade**		
10	29	19.3
11	66	44.0
12	55	36.7
**Ethnicity**		
Caucasian	113	75.3
Aboriginal and/or Torres Strait Islander	7	4.7
Asian	11	7.3
African	2	1.3
Middle Eastern	5	3.3
Prefer not to say	12	8.0
**Baseline perceptions of harms of smoking**		
Not at all harmful	1	0.7
Minimally harmful	6	4.0
Some harm expected	10	6.7
Quite harmful	20	13.3
Very harmful	113	75.3

Cronbach alpha for the Likert-scale questions was 0.89, indicating high internal consistency. Table 2 displays the results of the Friedman Test, showing the mean ranks (out of five) of each theme, and the *p*-values when comparing the mean ranks. Chi Square analysis showed that only gender effects were present, and the other demographic variables being not significant. Table 3 shows the proportional odds logistic regression model, including reference levels and points of significance. As an overall effect, participants perceived the warnings used on cigarette packaging and cigarette sticks as significantly (*p* < 0.001) more effective in preventing non-smokers from smoking, than in encouraging current smokers to quit with an odds ratio (OR) of 4.71 (95% confidence interval [CI]: 2.83–7.84).

**Table 2:** Mean ranks of interventions compared to current packaging warnings.

**Category**	**Mean Rank**	***P*-values**
**Perceived effectiveness in preventing non-smokers from smoking**		
Current warnings	2.99^bc^	–
Theme one (Mortality Statistics)	3.53^a^	<0.01^c^, <0.001^d^
Theme two (Health Condition Consequences)	2.94^ce^	<0.001^d^
Theme three (Social and Financial Consequences)	3.24^ae^	<0.001^d^
Theme four (Supportive Messages)	2.29^d^	<0.001^b^
**Perceived effectiveness in prompting current smokers to quit**		
Current warnings	2.57^c^	–
Theme one (Mortality Statistics)	3.25^a^	<0.001^c^
Theme two (Health Condition Consequences)	2.89^b^	<0.05^a^,
Theme three (Social and Financial Consequences)	3.32^a^	<0.001^c^, <0.05^b^
Theme four (Supportive Messages)	2.97^abc^	–

*Means with different superscripts are significantly different (p < 0.05) when adjusting for Bonferroni correction*.

**Table 3:** Proportional odds logistic regression model, with odds ratios for themes of cigarette stick warnings.

**Variable**	**Estimate**	**SE**	**Z Value**	**Odds ratio**	**95% Confidence Intervals**		***P-*value**
					**Lower**	**Upper**	
**DEMOGRAPHIC CHARACTERISTICS**							
Gender (Male = 0, Female = 1)	−0.22	0.39	0.57	0.80	0.37	1.72	0.566^NS^
**OVERALL THEME EFFECTIVENESS**							
Theme one warnings^b^ (MS)^∧^	1.02	0.26	3.90	2.77	1.67	4.62	< 0.001^***^
Theme two warnings^b^ (HCC)^∧^	−0.21	0.25	−0.83	0.81	0.50	1.32	0.405^NS^
Theme three warnings^b^ (SFC)^∧^	0.43	0.26	1.67	1.54	0.92	2.56	0.095^NS^
Theme four warnings^b^ (SM)^∧^	−1.26	0.26	−4.90	0.28	0.17	0.47	<0.001^***^
Effect on target smoking status (S = 0, *N* = 1)^a^	1.55	0.26	−5.94	4.71	2.83	7.84	<0.001^***^
**THEME EFFECTIVENESS ON TARGET SMOKING STATUS**							
Theme one: effect on smokers (*N* = 0, S = 1)^a^	0.04	0.36	0.12	1.04	0.51	2.11	0.908^NS^
Theme two: effect on smokers (*N* = 0, S = 1)^a^	0.55	0.36	1.54	1.73	0.86	3.51	0.124^NS^
Theme three: effect on smokers (*N* = 0, S = 1)^a^	0.64	0.36	1.76	1.90	0.94	3.84	0.079^NS^
Theme four: effect on smokers (*N* = 0, S = 1)^a^	1.89	0.37	5.18	6.62	3.21	13.67	<0.001^***^

a *N (Non-Smoker), S (Smoker)*,

*** *< 0.001, NS = Not significant*.

b *Reference level was the effectiveness of current packaging warnings*.

∧ *MS, Mortality Statistics; HCC, Health Condition Consequences; SFC, Social and Financial Consequences; SM; Supportive Messages*.

### Health warning effectiveness: cigarette packaging

Prior to being shown the interventional materials, nearly three-quarters (74.7%) of participants indicated that they had seen cigarette packaging. In response to the cigarette packaging warnings displayed, adolescents considered the warnings currently implemented on cigarette packaging to be somewhat effective in preventing non-smokers from smoking, though less so in prompting current smokers to quit (see Tables 2, 3). Most adolescents had strong personal opinions of the packaging warnings, describing them in the open-text comments as being “graphic,” “disgusting,” or “gross” in appearance, and considered them as effective in preventing themselves and other young people from smoking.

“*I thought it was quite shocking and would put people off smoking” (Male, 17 years), “It's gross and would definitely put me off smoking” (Male, 17 years), “Makes you never want to touch a cigarette” (Female, 16 years), “I think the packaging is enough of a reason not to smoke” (Female, 16 years)*.

However, participants also described their perceived shortcomings of current packaging warnings, with desensitization to the warnings being common amongst smokers, warnings that were too weak to cause emotional reactions, and poor relatability of depicted chronic diseases to teenagers being the most commonly cited.

“*People who smoke have been doing so for a long time and don't particularly care about the health risks” (Female, 17 years), “If someone wants to smoke they will just ignore the warnings” (Female, 16 years), “The packaging discourages me from smoking, though there are people who continue to smoke regardless of the packaging, which is sad” (Female, 17 years), “Should continue to be changed as people begin to get used to the disturbing images” (Male, 17 years), “The packaging seems to be directed towards adults, so it does not directly confront adolescents and young adults” (Male, 17 years)*.

### Health warning effectiveness: cigarette sticks

Amongst the four themes of cigarette-stick warnings displayed, theme one cigarette warnings describing mortality statistics (MS) from smoking were rated as the most effective (OR = 2.77; 95% CI: 1.67–4.62, *p* < 0.001) by adolescents, both in preventing non-smokers from smoking, and in encouraging current smokers to quit compared to current packaging warnings and the other themes presented. Female participants were significantly (χ^2^ = 7.743, *p* < 0.05) more likely to rate these warnings as effective in preventing non-smokers from smoking (61.5%) compared to males (48.1%). The cigarette describing the “minutes of life lost” was identified within the open-text comments as being the most effective warning in this theme, considered a novel and powerful message that would likely result in significant changes in smoking-related behaviors.

“*Smokers can actually see how much of their life they are losing” (Female, 16 years), “Seeing this as you smoke would discourage smoking and dull the experience” (Male, 17 years)*.

Theme two cigarettes warnings describing health condition consequences (HCC) of cigarette use were rated as similarly effective as current packaging warnings (OR = 0.81; 95%CI: 0.50–1.32, *p* = 0.405). The similarity between this theme and current packaging warnings was cited as an important limiting factor, with participants perceiving them as being a repetition of packaging warnings, likely resulting in the similar effectiveness ratings.

“*Everybody already knows smoking is bad and causes these diseases” (Female, 16 years), “The diseases mentioned are too common” (Female, 17 years), “People already know the effects, this won't do anything” (Female, 17 years)*.

Theme three cigarette warnings describing social and financial consequences (SFC) of cigarette use were also rated as similarly effective as current packaging warnings (OR = 1.54: 95% CI: 0.92–2.56, *p* = 0.095), though the cigarette stick depicting the financial costs of smoking was identified within the open-text comments as being notable and potentially effective.

“*Some people don't know or consider the long term effects other than health” (Male, 16 years), “Sadly people are now driven by money, so mentioning finances is effective” (Female, 17 years)*.

Overall, the theme four cigarette messages supporting smokers to quit (SM) were considered 0.28 times (95% CI: 0.17–0.47) less effective than current packaging warnings. However, in relation to smoking status, they were considered 6.62 times (95% CI: 3.21–13.67) more effective (*p* < 0.001) than current packaging warnings in prompting current smokers to quit. Open-text comments toward this theme was mixed, with participants acknowledging the need for positive messages which gave options for smokers to quit, though also believed that smokers would not be phased by this form of message in comparison to negative messages.

“*They would have to have the will to quit first, and this might tip them over the edge” (Female, 16 years), “The supportive messages can work for people who want to quit but haven't got the motivation” (Female, 16 years), “They know how bad smoking is and they can't stop, a bit of writing will not stop anything” (Male, 17 years), “A lot of people don't like being told what to do, especially if it involves their health” (Male, 17 years)*.

### Opinions of health warnings on tobacco products

Over three-quarters (78.7%) of participants either agreed or strongly agreed to the inclusion of health warnings and messages on individual cigarette sticks. Female participants were significantly more likely to agree (83.3%) compared to male (70.4%) participants (χ^2^ = 5.986, *p* = 0.05). Comments toward this question were generally positive, including by participants that had generally low ratings of the effectiveness of the cigarette stick warnings. The prolonged visibility of these warnings, and their effect on the esthetic of smoking were both identified as contributors to the potential effectiveness of this form of anti-tobacco intervention.

“*Being printed on the cigarette instead of the packet means it would be impossible not to notice” (Male, 16 years), “Seeing these warnings as you smoke or having other people see it would discourage smoking and dull the experience” (Male, 17 years), “It's better than messages on cigarette packets as smokers can actually think about what these messages mean whilst they are smoking” (Female, 16 years), “If at all possible this would be a huge step I think in reducing the rate of smokers in Australia, especially in the younger generations” (Female, 16 years), “Warnings scare people out of smoking and have had an impact on many smokers to stop, and prevented many non-smokers from starting” (Female, 17 years)*.

However, some noted that they would be ignored in a similar manner to current packaging warnings, especially by current smokers.

“*They might provoke thought though not make a complete difference” (Female, 16 years). “Would still probably suffer from loss of impact over time” (Female, 16 years)*.

## Discussion

This study aimed to first investigate the perceptions of adolescents on the effectiveness of current cigarette packaging warnings implemented in Australia, including their strengths and shortcomings. We also aimed to investigate their perceptions on the effectiveness of twelve cigarette sticks with attached text health warnings and messages compared to current cigarette packaging warnings, both in preventing non-smokers from smoking and encouraging current smokers to quit. We found that adolescents consider current packaging warnings as having retained some of their effectiveness in preventing non-smokers from smoking, though were relatively ineffective in prompting current smokers to quit. We also found that warnings describing the mortality statistics relating to tobacco use, and the financial consequences of smoking were considered novel and effective by adolescents.

The implementation of novel and cost-effective anti-tobacco interventions are theorized to be essential in reducing tobacco use and its associated morbidity and mortality (29). This is essential in particular for adolescents as a vulnerable population, as they have a limited understanding of addiction and other health consequences of tobacco use (7, 8), coupled with the increased potential for neural disruption of nicotine (4), and exposure to peer pressure and social tobacco experimentation (12). The specific and calculable losses of time (and to a lesser extent money), and mortality statistics of tobacco, compared to the threats of potential future ill health resulting from tobacco use may be perceived as more relatable, memorable, and effective. Previous research into the effectiveness of the minutes of life lost warning on cigarettes found it to have the lowest appeal ratings and greatest increase in quitting intentions (20, 21). Whilst no previous research has investigated the effectiveness of cigarette stick warnings describing the financial consequences of smoking, tax increases and the rising cost of legal tobacco products were described by participants in this study as well as elsewhere as being a strong motivator for quit attempts (30, 31). The general public, including smokers, have also been found to support tax increases of tobacco products, particularly if the revenue raised contributed to quit-smoking efforts (32). These findings and findings from similar research suggest that further research into warnings describing the minutes of life lost (20, 21) and mortality statistics from smoking, and specific financial consequences of smoking may foster reductions in tobacco use, in addition to those achieved through the current packaging warnings.

The shortcomings of current packaging warnings described by participants in this study were also similar to those identified in previous research (17, 18), and was supported by the similar Likert scale ratings for the theme two warnings describing specific health consequences of tobacco use. The gradual diminishing of warning effectiveness (17), and adolescent perceptions of personal imperviousness to the described health consequences (33, 34), require the use of warnings and messages that are novel, attract attention, and more relevant to adolescents. This may have contributed to the higher ratings of theme one and three warnings, which participants noted as being more novel and personable, as opposed to being common-knowledge or generic. Increasing the perceived threat of negative consequences related to tobacco use, and their perceived severity, and promoting cues to action and self-efficacy through the use of cigarette-stick warnings, may increase resistance to peer pressure and other trigger factors to smoking, which are often encountered during adolescence (19). As key elements of the Health Belief Model, we theorize that cigarette stick warnings achieve these effects through their own messages, as well as a cumulative or synergistic effect alongside cigarette packaging warnings, mass media campaigns, and other anti-tobacco interventions employed within the community. Shifting the balance of risks vs. benefits to emphasize the risks of tobacco use is therefore theorized to increase the likelihood of health promoting behaviors, which in the case of adolescents would ideally be a continuation of aversion toward tobacco products.

The high approval rating of including health warnings on cigarette sticks has been previously reported, including in the use of simple and well-recognized messages such as smoking kills (22–24). As the cigarette stick is the item consumed when smoking, it stands to reason that it should be made a component of the anti-tobacco arsenal and designed to be less attractive to reduce the appeal of smoking, in addition to unattractive and informative cigarette packaging, which may be hidden, discarded, or otherwise avoided by adolescents (21, 35). Though some smokers will either have no interest in quitting, and will not quit regardless of their awareness of the harms of smoking, these cigarette stick warnings may impact on risk taking behaviors of most adolescents.

Whilst this study found data supporting the effectiveness of cigarette stick warnings on adolescents, there are limitations to be considered when interpreting the results. The themes were presented in a standardized as opposed to a randomized manner, though all were presented on the same page, allowing students to adjust their Likert scale ratings easily. There was also a lack of blinding, which does not allow the effect of bias to be taken into account when interpreting the results. Due to the controlled, at-home environment of participation, we were ethically restricted from asking participants of their smoking status and experiences, and were unable to assess participant responses in real-world scenarios. Also, only private school and Catholic education students were enrolled, due to the overloading of Queensland public schools with research activities, potentially affecting the generalizability of the results to adolescents enrolled in public schools. Due to the online nature of the research, we were not able to gauge the response rate, nor the participants' level of understanding of the warnings shown, particularly of those describing health consequences of tobacco. Participants were also unable to hold cigarettes and experience tactile sensations which may have influenced their responses. Lastly, one of the warning images was misplaced into theme three (social and financial consequences of smoking), where its message was more akin to theme one, potentially affecting the theme three Likert ratings.

Based on the findings of this study, further research into the effectiveness of warnings on cigarette sticks, including which warnings are likely to elicit the greatest anti-tobacco effects on adolescents and potentially adults is a reasonable next step. To confirm the findings of this study and improve the generalisability of the results, a larger and more diverse cohort of school students is needed. The minutes of life lost message was rated as the most effective in this study and other studies utilizing this message (20, 21), and requires further investigation amongst a more diverse range of demographics to assess if it might be a universally-effective message.

## Conclusion

Reducing the prevalence of tobacco use, particularly amongst adolescents, is a major requirement for the future health of the global community, and a reduction in tobacco-attributable morbidity and mortality. Making the cigarette stick an educational tool alongside cigarette packaging interventions may further prevent the goal of the tobacco industry in recruiting the next generation of smokers. Cigarette stick warnings (such as describing the minutes of life lost per cigarette, and the financial consequences of smoking) which are novel and more relatable to viewers' appear to be the most effective. These interventions were strongly supported by adolescents in this study, who agreed that these warnings should be included on all cigarette sticks. Future effective warnings as suggested by adolescents in this study include the effects of smoking on children and other family members, and should be the focus for further research investigating the effectiveness of these warnings in preventing non-smokers from smoking, and encouraging current smokers to quit.

## Ethics statement

This study was carried out in accordance with the recommendations of the James Cook University Human Research Ethics Committee with written informed consent from all subjects. All subjects gave written informed consent in accordance with the Declaration of Helsinki.

## Author contributions

AD was responsible for the design of the intervention materials and conduct of the, and was also responsible for analysing the quantitative and qualitative data and writing the manuscript drafts. P-AT was responsible for reviewing and commenting on the manuscript drafts. BG assisted in the development of the intervention materials and revisions of the manuscript drafts. BM-A assisted in analysing the quantitative and qualitative data, and provided comments and revisions for the manuscript drafts.

### Conflict of interest statement

The authors declare that the research was conducted in the absence of any commercial or financial relationships that could be construed as a potential conflict of interest.
